# Exploring the Development of Chinese Digital Resources under Lightweight Deep Learning

**DOI:** 10.1155/2022/3759129

**Published:** 2022-06-29

**Authors:** Bai Song

**Affiliations:** School of Humanities and Social Sciences, Beijing Institute of Technology, Beijing 100081, China

## Abstract

From 2019, countries worldwide have been negatively affected by the corona virus disease 2019 (COVID-19) in all aspects of social life. The high-tech digital industry represented by emerging digital technologies is still vigorous, and correspondingly, the digital economy has become an important force to promote the stable recovery and re-prosperity of the national economy. The digital economy plays a memorable role in preventing and controlling COVID-19, the resumption of work and production, and the creation of new business formats and models. Urban big data (UBD) involves a wide range of dynamic and static data with high dimensions, but there are no mature and clear data classification and grading standards. Currently, it is urgent to strengthen the security protection of high-value datasets. Therefore, a UBD classification and grading method is proposed based on the lightweight (LWT) deep learning (DL) clustering algorithm. It uses a semi-intelligent path based on partial artificial to form data classification (DC) and hierarchical thesaurus, corpus, rule base, and model base. Subsequently, a big data analysis system is built for unstructured and structured data association analysis based on deep learning, spatiotemporal correlation, and big data technology to improve data value and adapt to multiscenario applications. Meanwhile, with the help of data and graphics processing tool Tableau, the present work analyzes the development status and existing problems of digital resources in China. The results show that although China's digital infrastructure is the top in the world, the trading infrastructure is still only 41.65 percentage points. This shows that China's digital economy still has a lot of room for growth in distribution and trading. The analysis of the ownership of data resources indicates that the scores of China's digital economy in accounting, privacy, and security are very low, only 2.4 points, 5.1 points, and 11 points, respectively. This study has solved the problems of distribution and trade in China's digital economy through research and put forward corresponding suggestions for the current development of China's digital economy market. Hence, a preliminary summary and suggestions are made on the development of China's data resources, to promote the open sharing of data, strengthen the management of data quality, activate the data resource market, strengthen data security, and enhance the vitality of the market economy.

## 1. Introduction

Throughout history, production factors are not static but constantly change according to the development of social patterns. More than 300 years ago, people would use land and labor as the password to obtain wealth. In the industrial era, the source of wealth has been replaced by sources, such as knowledge, capital, and technology. In the information era, data has become new production factors and marketing resources like the “oil” of the economy, having data resources means having development priorities. By optimizing their digital investments, business leaders and policymakers can be more competitive, productive, and bring the quality of life to people [1].

Since urban resources are not used indefinitely, the increase of idle resources will become a burden on urban development. Making full use of idle resources can improve resource utilization and reduce the construction cost of smart city. Smart city construction focuses on sharing, and maximizing the use of resources is the goal of smart city construction [2]. Urban construction is the centralized management of resources through a third-party platform. The third-party platform has problems, such as high cost and nonpublic transactions, which will directly affect the resource allocation of urban construction and become an obstacle to urban construction. The present work proposes an urban big data (UBD) classification and grading method based on lightweight (LWT) deep learning (DL) clustering algorithm, which makes the construction of smart city more secure [3].

In 2020, the added value of the digital economy in 47 countries in the world reached the US $32.6 trillion, with a year-on-year nominal growth of 3.0% and accounting for 43.7% of GDP. The digital economy in developed countries has a large scale and a high proportion [4]. In 2020, the scale has reached the US $24.4 trillion, with a year-on-year increase of 3.0% and accounting for 54.3% of GDP. The digital economy in developing countries is small, with a growth rate of the US $8.23 trillion, reached 3.1% in 2020 [5].

In 2020, the scale of China's digital eco economy reached 53,565 million USD, accounting for 38.6% of GDP, with a year-on-year nominal growth of 9.7% [6]. The Chinese government established data as a factor of production participating in social distribution for the first time in its latest policy announcement, and the state council further defined the direction and key reform tasks of data resources market system construction and stressed the need to accelerate the cultivation of data resources market [7]. Based on this, scholars have also carried out extensive research on data resources.

Starting from the supply of diversified data resources, Rui YANG [6] proposed to mine the needs of various industries for data application based on the analysis of data value chain and build a special department for the development of data resources market for unified planning and deployment.

Xiao [7] analyzed how to develop the market of data resources and build rules for governing data resources, proposed that the protection of individual privacy information should not be extreme, which will hinder the development of business and public utilities, and the use of data in business development cannot be based on the sacrifice of personal privacy.

Gang [8] analyzed the attributes of government data and believed that government data have the special attributes of public resources. It is not only an element of allocation in the market but also an element of social management and governance by the government. Government data should break through the resistance of various stakeholders, such as concept, technology, and security protection, and take “economic development leads to legal regulation” as the starting point to realize more effective and reasonable allocation of government data based on the market.

Jiang [9] analyzed the construction and development of the data resources market, mainly covering the confirmation of ownership, the definition of compliant transactions, and the construction of safeguard rules and regulations. She believed that multidimensional research should be carried out from different perspectives, combined with various relevant disciplines and research fields, to have a comprehensive concept about allocating data resources in the market.

Sun [10] believes that the government plays a very important role in the benign development of the data industry market and should plan and adjust the development of society and market as a manager and formulate corresponding policies.

We can see from the research and analysis of the above scholars, most studies only deconstruct the theory of data resource market from one aspect and fail to deeply explore the significance of cultivating data resources market, the overall environment of cultivating data resources market and how to promote the benign development of data resource market.

Deep learning technology has touched every corner of society and has profoundly impacted the way humans live and work today. Deep learning technology represented by convolution neural network (CNN) has developed rapidly in computer vision in recent years. It greatly surpasses traditional algorithms in image classification, object detection, and human gesture recognition [11]. Due to the increasing complexity of the neural network model, the traditional solution is to place the calculation in the cloud and then interconnect with the mobile terminal through the network. However, due to the limitation of network delay, network bandwidth, and no wireless network around the mobile terminal during data transmission, the development of this technology is very limited. In recent years, the computing power, storage capacity, and power consumption of mobile terminals have been greatly improved, which provides the basic conditions for the deployment of CNN in mobile terminals. At the same time, the large model size limits the embedding of CNN in the mobile phone software. The high computational complexity makes the mobile phone need to consume too much time to perform the forward operation on the neural network. This results in a poor user experience. Therefore, some scholars focus on how to simplify and optimize network models so that they can run smoothly on embedded devices with limited hardware conditions, and lightweight deep learning algorithms emerge as the times require [12].

Therefore, based on the practical significance and value purpose of cultivating the data resource market of China, this article makes an in-depth analysis of the data resources to provide reference for promoting the construction and development of the data resources market.

## 2. Purpose of Developing Data Resource Market

**Figure 1:**
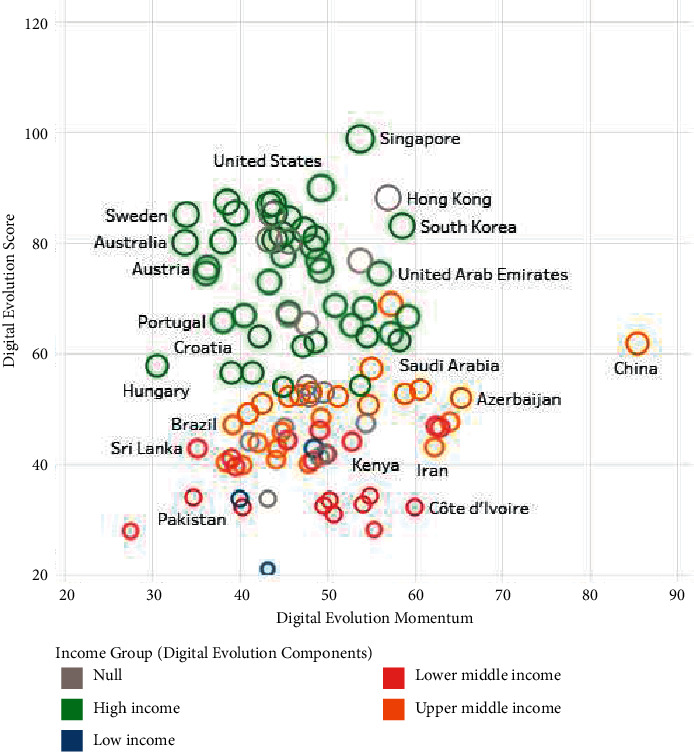
Digital state and momentum. Data source (DS): digital intelligence dashboard 2021, the Fletcher School TUFTS University.

According to the analysis of DII data in Figure 1, China's position in developing the global digital economy is very prominent and has very distinctive characteristics. Although China's development score in the digital economy is only above the medium level, its development potential is outstanding (near 90 scores). Based on this characteristic, this article will take China's digital economy as the starting point for analysis.

The existing digital economy shows strategic stability, and with appropriate facilitating policies and regulations, it will boost the upgrading of China's economic structure with breakthroughs in key technologies and achieve stable high growth. For the digital economy based on data resources, China has made great progress and occupies an important position globally, reaching 5.3 trillion, ranking second among the 47 economies in the world (Table 1). At the same time, the Chinese market is the most stand out regarding digital state and momentum (Table 2) due to its government having made many explorations in the development of digital resources. On June 3, 2021, the National Bureau of Statistics of China issued the statistical classification of the digital economy and its core industries (2021). From the two aspects of “digital industrialization” and “industrial digitization,” this paper determines the basic scope of the digital economy. It divides into five categories: digital product manufacturing industry, digital product service industry, digital technology application industry, digital factor driving industry, and digital efficiency improvement industry.

### 2.1. Major Economies in the World Already Explored the New Source of Economic Development and Enhance International Competitiveness

Around the world, data resources based on big data and digital economy have become the key focus of competition among countries. The rapid development of digital economy is both an opportunity and a challenge. Facing increasingly fierce international competition, countries all over the world have strongly realized the key significance of digital economy for promoting their own development and enable national economic development and social governance more effectively. The alliance and cooperation on the development of data resources have become a new focus of multilateral and bilateral cooperation all over the world. So far, more than 30 countries in the world have incorporated the application and development of data into their national development strategies. The United States is the first country in the world to develop digital economy, it is also the birthplace of data resources and the leader in large-scale practical application. The famous American economist, Richard R Nelson is the founder of the theory of economic growth and technological progress. The ideas in his papers and books have been cited by researchers in many fields. He argues that technological progress needs to be understood as an evolutionary process, and that technological progress depends much more on selection and learning than on prior calculation [13]. Therefore, technological progress is so much faster under competitive conditions than under monopoly or oligopoly conditions. An appropriate theory of economic growth must be explicitly linked to organizational change [14].

As early as 1998, the U.S. Department of Commerce publicly used the term “digital economy” to describe the changes brought by the change of information and data to the U.S. social economy and the world economy. Since the 1990s, the United States has gradually formulated and deployed its digital economy development strategy and data resources value mining by issuing a series of “digital economy development reports” such as “the emerging digital economy (1998).” The great potential of digital economy in promoting economic development is analyzed in detail, and the national digital economy development strategy is gradually created and deployed.

EU countries attach importance to close cooperation and strategic development relations between member states. The formulation and release of the “general data protection regulation” reconstructed the new pattern of data form development within the EU, broke the barriers between countries, established and improved the data development and data user protection mechanism, accelerated the confirmation of data resources, and protected the legitimate rights and interests of member states. The white paper “shaping Europe's digital future” and “European strategy for data” demonstrates the initiative of the EU in seizing the right to development in the field of digital economy.

The UK has created a detailed data governance development strategy and relevant policies to improve the efficiency of data governance. Policy documents include “open standards principles,” “open data: unleashing the potential,” and “UK data capability strategy: seizing the data opportunity.” Those strategies and public policies serve as specific practical guidelines to guide the UK's overall research and application of data resources, and promote the UK to a higher level in terms of legal system, data theory and data privacy protection.

The Japanese government has issued strategies to stimulate the potential of data innovation and build a systematic data analysis platform, so as to realize a systematic and intelligent scientific and technological innovation system. Actively encourage innovation, improve the national strategic development policy, creatively put forward the new theory of “society 5.0: a Japanese concept for a super intelligent Society” [15], strive to improve the national integrated innovation and comprehensive innovation ability, and actively give play to the role of the government in social governance and international cooperation and exchanges.

### 2.2. China Uses Digital Resources to Drive Industrial Upgrading and Promote High-Quality Economic Development

**Figure 2:**
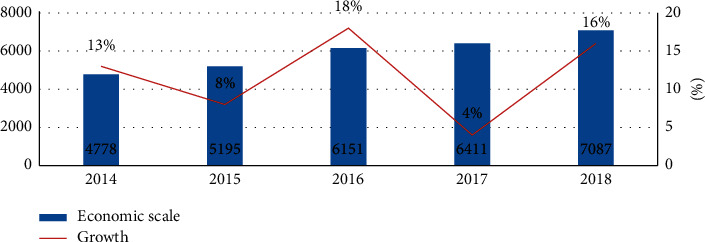
Scale of china's digital economy (trillion yuan, %). DS: China's information and communication research institute.

Cultivating and developing the data resource market will help realize and release the value of data resources, drive the continuous upgrading of industries, and promote the high-quality development of the national economy. The scale development of China's digital economy in recent years is shown in Figure 2.

Despite the increasingly fierce international competition, global economic environment has declined. However, the development of China's digital economy shows strong development potential driven by the empowerment of data resources. In 2019, the development scale of China's digital economy increased by 10% year-on-year, the economic scale has been close to 7087.8 billion yuan (Table 3), the growth pace is steady, and the economic scale has expanded steadily.

**Figure 3:**
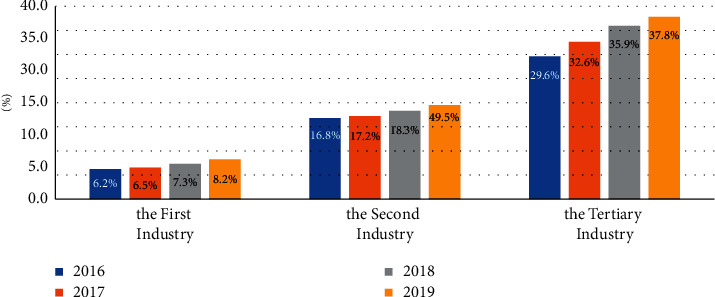
Contribution proportion of digital economy in industry. DS: China's information and communication research institute.

Improve the quality of national economic development. In the era of digital economy, data resources play a leading role in the digital transformation of economic subjects. Taking industrial Internet enterprises as an example, a fine management and product quality control system based on data technology has been gradually established, a cross departmental, cross industry, and cross link product quality supervision system based on big data has been gradually established, and a joint reward and punishment mechanism for enterprise product quality based on social credit data have been gradually promoted, which has brought changes in enterprise product quality and greatly improve the supply quality of enterprise products. The contribution proportion of digital economy in the industry in recent years is shown in Figure 3.

Furthermore, cultivating a perfect data resource market runs through the whole product life cycle, can fully realize the management and optimization of the whole product life cycle, and realize the industrial upgrading from low-end to high-end in China through all-round data governance at various stages of product development, listing, growth, maturity, and delisting, guide the transfer of low value-added industrial model (the first industry [16]) to cutting-edge industries dominated by high and new technology (the tertiary industry), and promote the transfer of economy to technology intensive, so as to promote the development of national economic quality (Table 4).

For the whole national economy, a perfect data resource market can comprehensively improve the efficiency of relevant economic activities focusing on production, collection, storage, processing, analysis, and service in the fields of infrastructure, data platform, and data application; activate businesses based on industrial data transaction services, data acquisition and processing, data security, resource management platform, and storage hardware facilities; form data applications covering finance, medical treatment, telecommunications, marketing, industry, tourism, agriculture, government, and space; and promote the reconstruction of production mode, industrial organization, and supply chain system [17]. Improve market resource allocation efficiency and production efficiency, and finally realize productivity reform.

Cultivating a mature data resource market can accelerate technology upgrading and create new jobs, so as to provide new impetus for economic development, continue to build new advantages in the development of digital economy, promote industrial integration, and help promote dynamic change. The new generation of information technology represented by cloud computing, big data, Internet of things, mobile Internet of Things, artificial intelligence (AI), and blockchain is the most active field of technological innovation and industrial development. With the gradual maturity of new digital technology, the market demand is constantly stimulated, and a large number of enterprises pour into form a new industry. At the same time, new technologies promote the formation of new business models. At present, the global epidemic situation is still severe, the international economic development situation is not optimistic, and the economic market is unstable and risky. However, the unstable epidemic environment provides a good soil for the development and prosperity of the digital economy, and the field of the digital economy shows a rapid development momentum. Different from traditional industries, data resources, as an emerging factor of production, are less affected by factors such as region and external environment. With the control of the epidemic situation in China, the economic operation is gradually back on track, and large-scale production resources are gradually flowing to emerging industries in the field of the digital economy. The new business format and new model [18] of the industry will lead the market to restore its original vitality.

### 2.3. Improve the Efficiency of National Modernization Governance

Cultivating the data resource market can not only ensure the efficient utilization of data value but also more effectively improve the national government's public management ability and national modern governance ability. Through cloud and big data technologies, government could comprehensively improve the digital level of decision-making, management, and services; harness digital technologies to create better outcomes for citizens; improve the modern governance level of the country and the government; and promote the sustainable development of the country and society.

First, the wide application of data resources promotes the transformation of national governance model and forms an information-oriented digital government. Every individual in the digital age is the publisher and owner of data. Society has become a real-time interactive information interaction space, and everyone can have an impact on social public policies. The magnanimity, diversity, and rapidity of big data make it difficult for any social organization or individual to monopolize data resources. Whether an organization, company, or individual can skillfully use big data has become an important basis for gaining the voice of social governance. This means that in the digital age, the government's public policy will actively or passively listen to the public's voice, and the subject of social governance should not and cannot be the single subject of the government, but should be integrated into multiple subjects, such as social organizations, markets, and the public, so as to facilitate the modern transformation of the social governance model; that is, from one-way governance of the state to two-way interaction between the state and society like private companies.

Second, the wide application of data resources promotes the openness and transparency of national governance process and improves governance efficiency. In the digital age, massive data are generated and obtained all the time. The data are open and fast flowing, which require the government to be open and transparent in the process of social governance. The whole process of government governance is supervised by the society, which helps the public to better understand and implement public policies, these new and powerful technological tools clearly have the potential to trans - form transparency. Due to the lack of participation of data resources, the traditional governance model leads to information asymmetry and low governance efficiency. Full data participation ensures the openness and transparency of social governance, which is feasible and operable.

Third, the wide application of data promotes the democratization and scientization of public policies and improves the modernization of national governance. In the digital age, the cost of public opinion expression and information transmission is low. Every citizen holds a microphone and speaks out through various channels at any time and forming massive and real-time data to a certain extent. Before formulating public policies, the state and the government can conduct a full sample survey to obtain rich and comprehensive sample data, so that the formulation of public policies can obtain a comprehensive, timely, and accurate factual premise.

Fourth, the wide application of data resources promotes the modern transformation of national governance and realizes people-centered and accurate governance. The application of data and its technology help national governance subjects obtain strong technical conditions in optimizing public service (PS) mechanism, improving PS mode, enriching PS content, improving PS level, and enhancing PS capacity.

## 3. The Basic Principle of LightWeight (LWT) DL Clustering Algorithm

### 3.1. *K*-Means Clustering (KMC) Algorithm

The goal of cluster analysis (CA) is to explore the cluster structure within the data samples, quantitatively express the various characteristics of the samples, describe the samples with specific indicators, and define the similarity measure between the samples. Clustering is to divide the data into multiple categories according to the internal similarity of the data, and each category becomes a cluster. As a research hotspot in the field of machine learning (ML) and data mining (DM), clustering is widely used in the fields of speech recognition (SR), image segmentation, biological information, web page information extraction (IE), AI, and so on. Common clustering algorithms, include KMC, mean shift clustering (MSC) algorithm, the DBSCAN clustering algorithm, the expectation-maximization (EM) algorithm using the Gaussian mixture model, and hierarchical clustering analysis (HCA) algorithm [18]. In particular, the KMC algorithm is the most used clustering algorithm because of its simple calculation and fast operation speed.

KMC algorithm is an unsupervised clustering algorithm. It is simple to implement and has a good clustering effect, so it is widely used. KMC algorithm has a simple principle, easy implementation, fast convergence speed, and excellent clustering effect. The algorithm has strong interpretability, and the main parameter to be adjusted is the number of clusters *K*. The main process of the KMC algorithm is as follows: firstly, given *N* pieces of data samples, the number of cluster categories *K* is manually determined from the original data set as the initial class center; secondly, these samples are divided into *K* clusters, the distance from each sample to the center *K* is calculated, and the new class center is iteratively calculated until a constant class center is determined [19]. The similarity between data samples in the same cluster is the largest, while the similarity between data samples between clusters is the smallest.

### 3.2. KMC Algorithm Based on LWT Deep Learning (DL)

Multisource heterogeneous data are collected by different data acquisition (DAS) methods. The attributes of data include hundreds of aspects, each of them are dimension of the data object. High-dimensional data about CA are an important problem to be solved in many application areas. The traditional clustering algorithm has some shortcomings in processing high-dimensional data and, thus, cannot get ideal results [20]. Therefore, given such voluminous amounts of data samples, it is challenging to ensure data safety and all data generation and applications are in a safe storage environment. Meanwhile, it is imperative to use data flow and database as storage systems to bind the access of data with the user's permission. In this way, users can only access the data within the scope of authorization to provide data training samples.

As a rule self-learning method based on multilayer neural network (NN), LWT DL can train massive amounts of data. Through big data-based feature learning, high-level abstract rules can be learned to serve the application needs of different fields. The idea of a DL-based KMC algorithm is to reduce the feature dimension before clustering operation. Firstly, NN can be used to reduce the dimension, then, the KMC algorithm can be used to cluster in low dimensional space. At present, the commonly used DL algorithms for dimension reduction (DR) include the Auto Encoder (AE) and Restricted Boltzmann Machine (RBM) [21].

**Figure 4:**
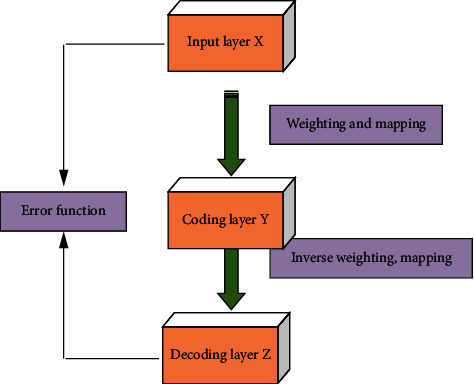
Feature DR of AE.

More precisely, AE is a self-supervised NN model, which learns the implicit features of input data through coding; whereas, the original input data are reconstructed from the newly learned features by decoding. The AE used for feature DR contains a three-layer NN structure, including input layer, coding layer, and decoding layer, as shown in Figure 4.

In AE, the three-layer NN can be used to reconstruct its input and learn the good representation of the input through its hidden layer. If the trained AE model outputs consistently with the input, a small number of neurons can suffice to represent the input data; that is, the model obtains the same representation of the input data with a smaller number of neurons. Based on this foundation, AE can be further subdivided into noise reduction (NR) AE, sparse AE, and variational AE, which can be more flexibly chosen for appropriate data formats to cluster them [22].

**Figure 5:**
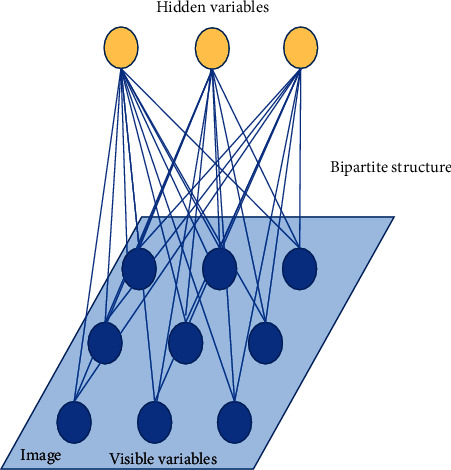
Overview of RBM.

RBM is a kind of two-layered stochastic neural networks (SNNs) model with symmetrical connection and no self-feedback. There is a full connection between layers and no connection within layers. Besides, RBM is much similar to the AE, only with a three-layered architecture rather than a two-layered structure [23]. Especially, RBM also uses its hidden layer for the feature representation of the original data.  Figure 5 gives an overview of the RBM:

## 4. Development Status of Data Resource Value

Although the National Bureau of Statistics promulgated the statistical classification of the digital economy and its core industries in June 2021, which determined the basic scope of digital economy and data resources, have been fully valued. UBD classification is an important form of data in-depth mining, which plays an important role in data security protection and effective utilization. Although China has issued and implemented relevant data classification (DC) systems, laws, and regulations. UBD-oriented DC still lacks mature and clear standards, relies more on workforce, is highly restricted by personnel, and has low data processing efficiency. Thus, relevant industrial personnel is required to have experience in the industry, business, and information security to improve the accuracy of DC.

### 4.1. Construction of Big Data Analysis System

#### 4.1.1. Analysis of System Application Requirements

The application requirements of the system include three functions: association query, data collision analysis, and data statistics. The query and retrieval function focuses on the file information and timing log information formed by the association between image entities and attributes and provides the functions of searching files by pictures and graphs by attributes, as well as the search of collected data logs and the query of timing track data. Log search finds log data according to the collection device identification, attribute information, and time range. The timing track data searches the time series log data belonging to the same entity according to the entity ID (identification), attribute, and time range, and forms the track description and map display.

Data collision analysis includes spatiotemporal data collision analysis and mixed trajectory analysis. Taking the image, attribute, and time as the collision range, it obtains the hit data, displays them through the map, and supports filtering and retrieval according to the time range, area, and acquisition front-end. Mixed trajectory adjoint analysis selects a specific time period and region to analyze the adjoint situation of entities and attributes in the region [24].

Data statistical analysis includes collection volume statistics, file statistics, collection point statistics, and research entity attribute correlation confidence. All accessed collected data shall be counted by time period according to the data category, including the number of graphs, the number of attributes, the number of files formed, the number of front-end collection points, and the amount of collection. The entity attribute correlation confidence is based on the entity attribute correlation times. Through the association rate algorithm, the association confidence is given according to the value interval.

#### 4.1.2. System Architecture Design

**Figure 6:**
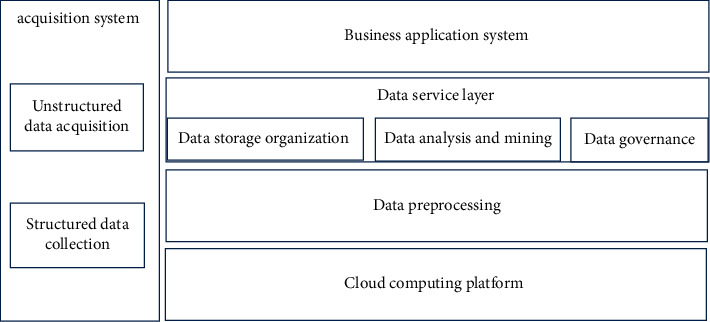
System architecture.

The big data analysis system is divided into a front-end acquisition system and a backend data center. The system architecture is presented in Figure 6.

The acquisition system is the front-end sensing equipment deployed in various indoor and outdoor venues, such as roads, parks, squares, and other outdoor places, as well as access control, corridors, and other indoor places. The acquisition system has the ability of video image acquisition and electromagnetic signal acquisition and can output unstructured data and structured data. The number of front-end equipment varies greatly according to the actual situation, ranging from hundreds to thousands. The amount of data collected increases with the increase of the number of front-end devices. The capacity of the backend data center can be extended horizontally and expanded dynamically.

The backend data center is built based on the cloud computing platform, and the data service layer and business application system are built based on the storage and computing capabilities provided by the cloud computing platform. The data service layer supports the business application system to carry out relevant businesses through data services [25, 26].

The data service layer includes several subsystems: data preprocessing, data storage and organization, data analysis and mining, and data governance. Data preprocessing includes data cleaning, entity unification, entity filing, attribute filing, and comparison identification module. The first data cleaning module provides the function of data de-duplication in a period of time, the entity unification module provides data recognition and data analysis from the front-end of the graph and then returns to the ID. The entity filing module creates an entity file according to the unique feature ID. The attribute filing module creates files according to attributes, and the comparison identification module can compare the source data with the preset comparison conditions, entity IDS, attributes, and their combination rules, and identify the hit data according to the rules. The data storage and organization subsystem includes log storage, file storage, and association relationship storage modules, which provide the storage functions of logs, files, and association relationships. The data analysis and mining subsystem include entity aggregation, entity attribute correlation, and classification statistics modules. Data governance includes resource directory and data service module.

The business application system provides external functional applications. Based on the ability of the data service layer, it provides log query, file query, association analysis, map display, relationship display, collection equipment management, operation and maintenance, and other functions.

### 4.2. Measurement Indicators and Development Status of Digital Economy

A comprehensive and in-depth discussion on the development of China's digital economy cannot avoid the CCID digital economy development white paper regularly issued by China Electronic Information Industry Development Research Institute (CCID group, a scientific research institution directly under the ministry of industry and information technology). DEDI (China Digital Economy Development Index) calculated by CCID group based on the indicators of various dimensions of digital economy and using the method of data statistics, is the core index for analyzing the development of digital economy in China and various industries in various regions.

### 4.3. Data Analysis and Mining Algorithm and Implementation

The data analysis and mining subsystem includes three core functions: entity clustering, entity attribute correlation, and multidimensional statistics. Entity clustering use the DL technology for entity recognition and feature comparison. Entity recognition identifies the entities in the data through a specific deep neural network. According to business needs, it can be a person, car, object, character, voiceprint, or other conceptual entity in the data. The essence of feature comparison is a multiclassification problem of *N*. The trained neural network is used to extract the image depth features, and the nearest neighbor classifier is used to identify by comparing the distance between the depth features. Euclidean distance or cosine similarity is often used to measure feature distance. For example, the specific entity features of image *X*_*i*_ and *X*_*j*_ are *F*(*X*_*i*_) and *F*(*X*_*j*_), respectively. When the distance between features is within the preset threshold *t*, that is:

(1) $$\begin{array}{c} D\left[F\left(X_{i}\right)-F\left(X_{j}\right)\right]⩽t. \end{array}$$

The two images are from the same entity. Large scale data retrieval based on feature vector faces the problem of low retrieval performance caused by large number of features and high feature dimension. The system speeds up the search by building a high-dimensional feature index. The entity aggregation file searches and compares the given image with the existing image in the system. If successfully matched, the unique ID is returned. If the matching fails, it is a new entity, and then a unique ID is assigned and stored in the system.

Entity attribute correlation realizes the association of entities and attributes in the same time and space. Whether they belong to the same collection space is judged by the device group ID, and a certain time interval is counted as the same collection time. In the actual scene of the system, generally, less than 10 pictures are collected by a device group within 2 min, but the collected attribute data are in the order of hundreds. If the picture entity and attribute appear together once in these data, it is recorded as a correlation. The number of correlations and attribute occurrences are counted according to the device group, and the number of correlations is *N*_*g*_={*N*_1_, *N*_2_,…, *N*_*n*_}, where *N*_1_ indicates the number of correlations in device group 1; number of occurrences of correlations is expressed as *M*_*g*_={*M*_1_, *M*_2_,…, *M*_*n*_}, where *M*_1_ indicates the number of occurrences of the correlations in device group 1.

The correlation rate *P* is calculated as

(2) $$\begin{array}{c} P=\sum_{g=1}^{n}N_{g}÷\sum_{g=1}^{n+4}M_{g}. \end{array}$$

The correlation rate *P* ranges in [0, 1]. The association rate of entity attribute has two directions. First, the association rate from entity to attribute, that is, the above calculation method. Second, the association rate from attribute to entity, which can be obtained by taking the ratio of association times to entity occurrence times according to the above method.

Multidimensional statistics include collected data statistics, log statistics, picture statistics, equipment statistics, and file statistics with the device group ID as the statistical dimension. The statistical data are calculated in the process of streaming processing, and the results are written into the relational database.

### 4.4. Calculation Method of EDI Index

In order to eliminate the problem of different index units, the data are dimensionless first. Different dimensionless methods are selected according to different indicator data types.

That is, the original value of each evaluation index is *x* (*i* is the indicator object and *j* is the indicator number), the dimensionless value *Z*_*ij*_, and the calculation matrix of index *j* is *x*_*ij*_.

Processing of numerical indicators: in order to avoid the imbalance of indicator differentiation caused by too large difference of original values, the logarithm method is used to dimensionless the indicators:

(3) $$\begin{array}{c} z_{ij}=\left(\ln\left(1+\frac{x_{ij}}{x_{ji}}\right)\right)\times 50. \end{array}$$

Processing of index indicators: such indicators only need to normalize the data:

(4) $$\begin{array}{c} z_{ij}=\frac{x_{ij}}{x_{j}}\times 50. \end{array}$$

Calculation of base value: the base value of the index system selects the average value of 31 provinces and cities in China.

The index weight is determined by expert scoring method. The expert group scores the weight of the secondary indicators in the evaluation system, and the total weight score of the indicator system at all levels is 100. The final weight of the index is the average value scored by experts. The weighted average method is adopted for the calculation of indexes at all level of each object:

(5) $$\begin{array}{c} z_{i}=\frac{\sum \lambda_{i}Z_{ij}}{\sum \lambda_{j}}. \end{array}$$

### 4.5. Granger Causality Test between DEDI and GDP

After confirming the calculation method of DEDI, we will use Granger causality test to verify the relationship between GDP and DEDI. Granger causality test is a measurement method widely used by economists. It judges whether there is a causal relationship between *Y* and *X* by comparing “knowing all the information at the previous time and the probability distribution of *X* at this time” and “knowing all the information except *y* at the previous time and the probability distribution of *X* at this time.”

In this analysis, we assigned the GDP and DEDI of Q4 in 2017 to Q3 in 2021 as *X* and *Y*, respectively, created the Granger causality test in EViews software quarterly, and set the lags value to 2. The specific *X* and *Y* values are as follows:

The results of the Granger causality test are as follows:

According to Table 5, GDP and DEDI are cause and affect each other and have a high correlation. It can be inferred that the development of digital resources plays a very important role in promoting the growth of national economy. At the same time, the development of national economy can also drive the further development of digital resources.

#### 4.5.1. The Increase of DEDI

**Figure 7:**
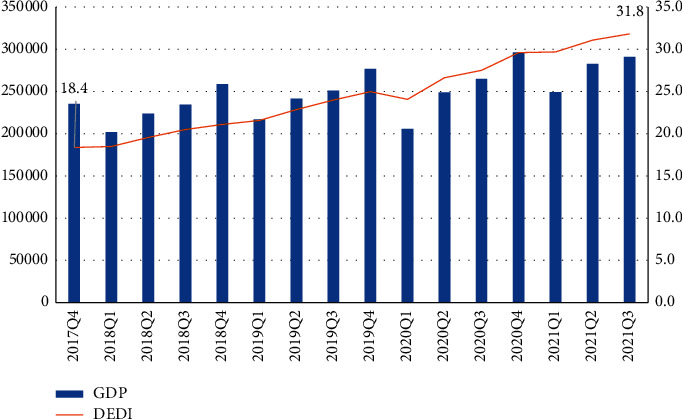
The increase of DEDI. DS: china national bureau of statistics.

From the data I collected before, Figure 7 reveals that China's DEDI index has achieved rapid growth from 2017 to 2021, which also shows that China's digital resources have made great progress in recent years.

However, in addition to these obvious phenomena, this paper also wants to elaborate on the problems existing in the development of China's digital resources through DEDI and other qualitative analyses.

## 5. Construction of UBD Classification and Grading Method

### 5.1. UBD Classification and Grading System

The UBD classification is to accurately describe the types of urban basic data through multidimensional data features, scientifically manage UBD, correctly develop and utilize data according to their categories, and mine and utilize data value more effectively. Comparatively, UBD grading refers to assigning different sensitivity levels to data tables, data fields, and data records according to the data sensitivity; of these, data tables and data fields are graded according to the sensitivity of their attributes, while data records (including database and its encryption measures) are graded according to the sensitivity involved in their contents (usually according to the influence of the data record object); data grading is the first step to implement data isolation and data protection. A single data item or data set (including multiple data items) is divided into several levels according to specific principles, methods, and processes, or, in other words, data security can be divided into four levels according to the impact object, impact scope, and impact degree caused by data leakage, tampering, and abuse.

The specific description of the data security level is as follows: (1) level I: data leakage, tampering, and abuse will have a general impact on a single organ, PS institutions, other institutions, and natural persons. (2) Level II: data leakage, tampering, and abuse will have a serious impact on a single other institution or a general impact on multiple other institutions and natural persons. (3) Level III: data leakage, tampering, and abuse will have a serious impact on a single organ, PS institutions, and natural persons, or a general impact on multiple organs and PS institutions. (4) Level IV: data leakage, tampering, and abuse will have a particularly serious impact on a single organ, PS institutions, other institutions, and natural persons, or on multiple organs, PS institutions, other institutions, and natural persons.

The purpose of UBD grading is to determine the sensitivity of different types of data to provide support for data sharing and opening strategies. UBD classification is the intermediate process of connection management and technology. From the perspective of management, the classification and grading of data can be combined with the management system of safeguard measures and operation systems, thereby strengthening the enforceability of the implementation of the management system; technically different security protection can be implemented according to different data security levels. For example, fine-grained rule control and data encryption are required for high-level data, while the one-way audit is only required for low-level data.

#### 5.1.1. DL Clustering Algorithm

As a product of ML development, DL technology has been most widely used in NLP, SR, and image processing (IP) in recent years. To apply DL clustering algorithm to classify and grade UBD, it is necessary to use natural language understanding (NLU) technology to segment and label UBD; finally, UBD classification and grading thesaurus and corpus are constructed, together with UBD classification and grading rules; in this way, the automation and intelligence of UBD classification and grading are realized.

NLP is an interdisciplinary science of linguistics and AI, which aims to enable computers to “understand” human language. The key technologies include word segmentation (WS), parts of speech (POS) tagging, Named Entity Recognition (NER), syntactic analysis, keyphrase extraction (KE), text classification (TC), automatic summarization (AS), and information retrieval (IR). With the development of ML shallow models, such as logistic regression, some breakthroughs have been made in the field of NLP, but there are still obvious deficiencies in word sense disambiguation (WSD) and NLU.

In terms of algorithms, as the pioneer of the DL algorithm in the field of NLP, word vector has a wide range of application scenarios and has achieved excellent results in machine translation (MT) and emotion analytics (EA). Its basic idea is to convert words in natural language into machine-understandable dense vectors with limited dimensions. So far, DL-based NLP has seen broad applications, such as TC, MT, intelligent question answering, recommendation system (RS), and chatterbots.

#### 5.1.2. DC and Grading Method Architecture

**Figure 8:**
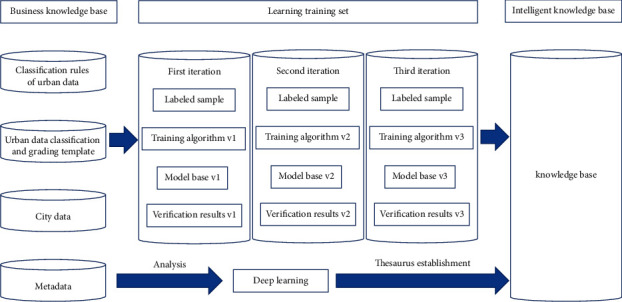
DC and grading method architecture.

The designed DC and grading method architecture designed is unfolded in Figure 8:

The application of NLP technology based on LTW DL clustering algorithm in UBD classification and grading is a semi-intelligent path based on partial manual labor. More precisely, in the early data training set, it manually segments and labels the field information of each data resource to form a DC and grading thesaurus, corpus, rule base, and model base.

The LWT DL clustering algorithm is used to continuously carry out iterative training and learning so that the machine can automatically find sensitive data, build a “rule engine” to drive all links of the whole process of classification and grading (including management rules and technical rules), and strengthen the intelligence of UBD classification and grading under rule control. In this way, the LWT DL clustering algorithm adapts, updates, and maintains the rules of DC and grading, regularly verifies the rationality of the rules through man-machine combination, dynamically improves the rule base, iteratively updates with the changes of the rules, meets the requirements of flexible adaptation and management of the rules, and finally, realizes the automation and intelligence of UBD classification and grading.

### 5.2. Limiting Factors for the Development of Digital Resources in China

#### 5.2.1. Data Technology and Infrastructure Construction Standards Are Not Unified, and the Available Data Resources Are Still Insufficient

**Figure 9:**
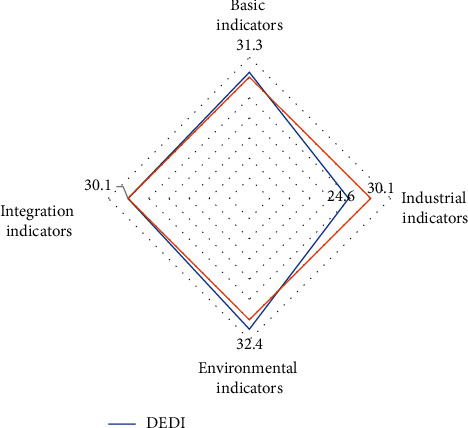
DEDI values of four primary indicators. DS: CCID consultant 2020.

From the four indicators of DEDI: basic indicators, industrial indicators, environmental indicators and integration indicators, the DEDI of industrial indicators in 2020 is far lower than the average value of primary indicators, as shown in Figure 9:

This shows that the application of data resources in the industry is not sufficient. Enterprises have constraints on the utilization, creation, and exchange of data resources. One of the main reasons is that data technology and infrastructure construction standards are not unified, resulting in available data resources are still insufficient. Specifically:

With the popularity of the Internet, sensor networks and mobile networks, heterogeneous data from different sources have accumulated rapidly, forming a massive data set. Because these DSs are diverse and multimodal, the uncertainty and diversity of multisource and multimodal will inevitably lead to differences in data quality and seriously affect the availability of data. This heterogeneity makes optional technical standards only cause market conflict and confusion. Therefore, in order to judge the accuracy, consistency, integrity, and timeliness of data, it is necessary to standardize data resources with mandatory technical standards and recommended technical standards, and the formulation of data technical standards needs to be promoted by the government. However, as the data of industry regulatory standards, its size can be regarded as the fulcrum of an industry. Each new standard release and old standard adjustment will change the existing interest pattern of an industry. The more the government deregulates the technical standards and delays the promulgation of the technical standards, the more profitable the enterprises will be. The government should give further play to its guiding role in the market, speed up the formulation of data technology standards in various fields to promote the standardized flow of data resources.

If the technical standard of data resources is the software for the construction of data resources market, the construction of data infrastructure is an indispensable hardware. To become a production resource, data needs two important technical foundations.

First, the volume of data should be large. In fact, in the early 1950s, with the maturity of magnetic core memory technology, data information can be stored effectively, but until 2008, data does not have the role of real production resources. The fundamental reason is that the volume of data has been relatively small, so data can only improve production efficiency through “digital industrialization” in some high-tech industries, but it has little effect in other industries. However, after 2008, with the advent and popularization of smart phones, the massive data from smart phone users increased sharply. Then, with the rapid development of cloud computing technology, the massive data scale, and rapid data flow transfer data really became a multiplier that can radiate all production departments and organically combine with other resource markets.

Second, data resources are processed data. In the era of big data, data does not completely depend its quantity to become a production resource, because data has significant heterogeneity in terms of its technical attributes. Due to different purposes of users and different data collection objects, the value of each bit of data is completely different. Data can only be digitized, cleaned, analyzed, modeled, visualized and other operations around the basic resources of big data according to different user needs. Only after it is transformed into information can it become a productive resource that can be used and its value can be measured, rather than directly put into the production field such as land, technology, and labor force. Therefore, the data that has not been digitized is not big data. As a production resource, big data is digitized and intelligent data.

Based on this background, the data infrastructure white paper 2019 puts forward that the difficult problems of data infrastructure construction focus on three problems: data storage, flow, and poor usage.

Firstly, economic development has entered the era of digital economy, and one of the basic changes is the exponential growth of data. According to Huawei's global industry outlook, the amount of newly generated data in the world will grow rapidly from 32.5zb in 2018 to 180zb in 2025. Because the storage system is still a traditional architecture and cost, less than 2% of the current enterprise data is saved, and the problem of “unable to save” data will become more and more serious.

Secondly, the huge volume of data comes not only from the absolute growth in the number of data supply sources but also from the flow of data. The value of isolated data and “flowing” data is not unified. Only the data breaking through “barriers” can stimulate the potential value of data. However, due to the limitations of technology and policy, only 10% of the data saved by enterprises can be analyzed. Data island, diversity equipment, and business migration have become the main bottleneck of data “immobility.”

Finally, the data resources are different from any other resources in the past. Only processed data can effectively promote the development of productivity. “In the era of big data, a typical analysis business usually needs cross platform data collaboration. If the data analysis link is lengthy, once there is a problem, it needs the “six-party talks” to locate, which cannot ensure the stable and high availability of data supply, let alone achieve efficient data fusion analysis.” [24] Therefore, the realization of data collaborative processing is also one of the difficulties in the construction of data infrastructure.

#### 5.2.2. The Distribution Subject Is Not Clear, and Distribution Mechanism Is Unfair

**Figure 10:**
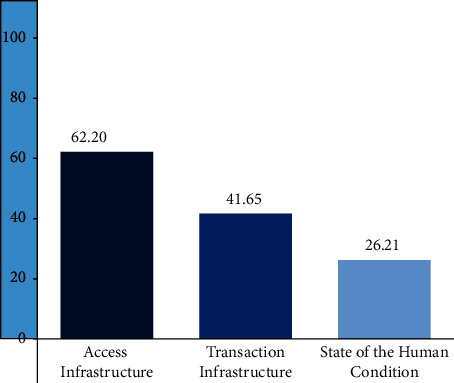
DII of China in infrastructure. DS: digital intelligence dashboard 2021, the fletcher school TUFTS University.

At present, there is no in-depth research on the distribution subject of data resources in China. There is still a vague definition of the distribution subject of data resources, mainly in the following aspects, as Figure 10:

Figure 10 suggests that although China's digital infrastructure is at the world's top level, the transaction infrastructure is still only 41.65 points, indicating that China's digital economy still has a large room for growth in distribution and transaction.

Right now, enterprises or digital platforms are the main distribution controllers of data production resources. Relevant enterprises spend a lot of costs in the process of data collection, screening, mining, and development, which makes pure and isolated personal data become big data with great force, and they think they should become the main body of data resource allocation.

The leading enterprises in the field of data resource application are basically concentrated in China's developed regions, resulting in a great gap in the development of digital resources among different groups and regions, which means that users in different regions enjoy great differences in the development dividends of data resources:

In this case, some scholars believe that ordinary users as DSs should become the main controllers of data resource allocation. In their view, the source of general data can neither be the arbitrary creation of digital platforms, nor the labor products of workers engaged in social production. Data are the result of every search, purchase, and entertainment behavior of individuals living in the digital age when using network applications and intelligent services.

At the same time, we should pay attention to the distribution subject of data production resources ignores the technical staff engaged in digital labor. In the view of entrepreneurs and ordinary people, technical staff are neither the source users of data nor the possessors of data. They should not participate in the contribution distribution of data resources at all. Even the technicians engaged in digital labor themselves cannot see the importance of their labor to give full play to the value of data.

### 5.3. The Ecological System of Data Resources Industry Is Not Perfect and Lacks a Reasonable and Effective Supervision Mechanism

Overall, the construction of China's data resources industry ecosystem is still in its infancy. In the integrated wave of production, learning and research, the development of data resources lags the renewal speed of the innovation chain. There is not only a large gap in the development level of digital economy but also great differences in the services and supervision mechanisms of local governments for digital resources, as shown in Tables 6 and 7.

The collection, transmission, and aggregation activities involved in data transactions are becoming more and more frequent. There is an urgent need to establish a participation mechanism in the whole process, including regulators and social organizations. From the perspective of the industry, the enterprise is still a weak link in the classified management ability of data. Local governments still need to give full play to their governance influence and driving force in digital resources.

#### 5.3.1. The Characteristics of Data Resources Determine the Real Dilemma of Data Ownership

In addition to the above summary of problems obtained from the sorting and analysis of DEDI data, through in-depth understanding and research on some industries and enterprises, combined with the theoretical analysis of economics, I found that there are great obstacles in the development of data ownership in China's digital resources.

**Figure 11:**
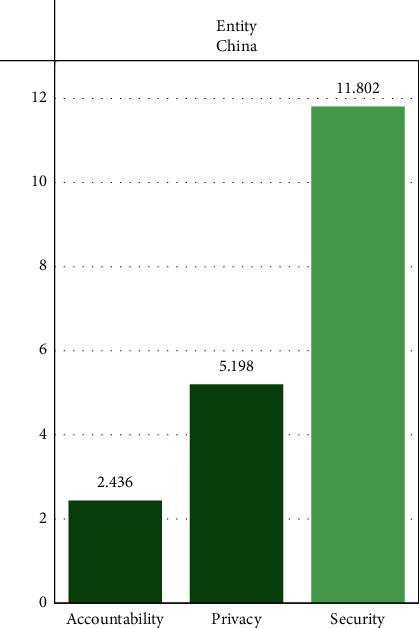
DII of China in data ownership. DS: digital intelligence dashboard 2021, the fletcher school TUFTS University.

The ownership of data assets plays a decisive role in the distribution of data value interests, the division of data quality, and security responsibilities. It is one of the core factors in the formation of digital economic income system and promotes the marketization of digital elements. The problem of data ownership originates from the progress of social production and the development of digital technology. At the beginning of data generation, there was no problem of data ownership and protection. Data were regarded as a public product and could be obtained and used by any market subject. After the rise of big data technology, processed data became an important source of value and played an important role in the market subject. In the process of data accumulation, data collection, processing, analysis, and trading are becoming more and more specialized, showing huge market value. Due to the scarcity and value of data, it continues to lead to conflicts between subjects in each link of the data industry chain to achieve the balance between data protection and utilization. It is necessary to clarify who owns the data, the collector and the holder, or the individuals and organizations that generate data subjects, and on this basis, clarify the data ownership. The analysis of the basic characteristics of data resources is the primary basis for the establishment of the right confirmation framework. The DII of data ownership in China is shown in Figure 11.

DII's analysis of data resource ownership can clearly show us the problems faced by China's digital economy in accounting, privacy, and security. The scores of these three items are very low, only 2.4, 5.1, and 11. In the following chapters, we will describe in detail the problems of data ownership in combination with digital resources and China's economic characteristics.

First, data are intangible, which makes them difficult to be completely occupied by any specific subject. They have the characteristics of nonexclusivity and are not suitable for the current requirements of the object of real right protection. As we all know, the object of real right is mainly aimed at tangible things, including movable and immovable property. The characteristics of the data itself cannot be directly incorporated into the traditional real right protection framework, and the current real right law is also difficult to determine the corresponding ownership for the data. In the era of digital economy, the data itself lacks clear exclusivity. It will not be collected by only one subject; any subject can collect and occupy the data in different ways. Therefore, the theories of data ownership and property rights discussed in the current property law are not suitable for establishing the ownership of data assets.

Second, the data are difficult to clarify their “originality” value, which is only the existence of an objective fact, which makes the data collected and stored by production enterprises as market subjects difficult to be protected by traditional intellectual property law at this stage. The current intellectual property law arranges the creative works according to the clue of time order, establishes their specificity, and protects them, but it is difficult to apply to data compilation works. The current copyright law only protects the data arrangement, not the data itself. The economic value of the data itself cannot become the object of protection of the copyright law.

Third, data generation depends on personal actions, and the personal information formed in actions has a broader connotation. Digitized personal information includes not only the category of personal privacy but also behavioral concepts involving personal value orientation, dignity, freedom, and other basic rights, such as political position, economic situation, interests, and hobbies. Therefore, the right to personal information goes beyond the scope of the right to privacy. In this sense, the traditional protection of the right to privacy has been difficult to interpret digital personal information, the protection specifications of data and personal information need to be reproduced. Fourth, in the data environment, it is difficult to identify the “confidentiality” of trade secrets that reflect the core competitiveness of market subjects, and the “management” identification is affected by cloud services. The openness of the data itself blurs the nonpublic and nonpublic characteristics of trade secrets, which makes the identification of the elements of trade secrets uncertain.

Due to the permanent preservation of data, it has a long derivative chain, and complex and diverse equity subjects are involved in the whole life cycle of data processing, and it is difficult to carry out a single authorization. Therefore, from the technical attribute, it is difficult to establish an exclusive right system arrangement to stimulate the supply and effective use of data. Moreover, in the production chain of data resources, data suppliers are naturally in a weak position. Without clear legal provisions and relevant regulatory mechanisms, the interests of data suppliers are difficult to guarantee, and the vitality of DSs will be lost in the long run. Therefore, how to balance the efficiency of data production and the rights and privacy of data providers has become an important problem in the flow of data resources. As some scholars pointed out, from practical experience, countries around the world have not adopted the model of “clarifying property rights first and then promoting data capitalization.” If the value of the data cannot be reflected, then all stakeholders related to the data do not have the enthusiasm to participate in the confirmation negotiation, and it will be more difficult to truly realize the clarity of data property rights. At present, the data security law of the People's Republic of China has just been promulgated, and the personal information protection law has not been formulated. Relevant provisions on data security and personal information protection are still being explored. The relevant provisions are not specific and clear enough to be directly applicable to law enforcement, cannot meet the development needs of new business forms, and cannot meet new problems such as data rights protection and data trading. Secondly, the subject of data property right is not clear, and there is “data monopoly.” As the creators of massive data resources, people will form many isolated and pure personal data in the process of using search engines, Taobao shopping, and online social networking. These seemingly useless user data will have immeasurable commercial value, political value, and strategic value after being processed by modern technical tools. However, since the subject of data property rights has not been scientifically and reasonably defined, these data are collected and occupied by domestic big digital platforms. The recent data leakage incidents “DIDI” [25] can make us clearly realize that monopoly and mobile Internet will make massive data fully concentrated in the hands of one enterprise. Without effective supervision and control, it is easy to trigger the disorderly expansion of capital and lead to the development threat of the country and society.

#### 5.3.2. The Data Security Mechanism Is Backward, Forming the “Achille's Heel”

After expounding the problems existing among each stakeholder, I would also like to emphasize a major problem facing China's digital resource market: the data security mechanism, as already mentioned in Figure 7.

At present, this potential safety hazard is mainly reflected in the following two aspects. First, data disclosure may be easier. With the advent of the 5g era, the digital economy ushers in a period of vigorous development. More advanced sensors will be used to collect, screen, and process data, and more complex terminals will connect different areas of life to truly realize the interconnection of all things. At the same time, this means that the user data of ordinary people will be easier to obtain, and the risk of data leakage will be greater. Secondly, the problem of data abuse is emerging one after another. In the digital age, the hidden danger of data security not only comes from the theft of external criminals but also the commercial behavior of abusing data on some digital platforms. Since entering the Internet era, the problem of data abuse has existed for a long time. However, in the past, the data leaked by people was very limited, including only user data such as mobile phone number and personal e-mail. It is usually easier to prevent, and the damage to people's rights and interests is relatively small. However, with the development of modern technology tools, user data have been involved in various fields of personal life, such as clothing, food, housing, and transportation. These extensive user data will be put into different business fields by digital platforms, which will cause great damage to people's rights and interests.

## 6. Suggestions on the Development of China's Digital Resource Market

Based on the analysis of China's data resource market in the previous chapter, this paper will suggest how to cultivate the digital resource market based on the existing problems and future development direction.

In order to achieve higher quality development of digital economy, we need to lay out the important work of data resource market cultivation with a forward-looking development perspective, such as professional talent reserve, improvement of laws and regulations on information protection and privacy policies, research and development of innovative technologies, and new models of data disclosure and sharing. These are new models and new actions to build a new business form of the digital economy in the new era. To realize the maximum utilization of data value and promote the transformation and driving effect of the digital economy, we can think and work from four aspects: data open sharing, data quality management, data transaction circulation, and data risk regulation.

### 6.1. Promote Open Sharing and Enrich the Source Supply of Data Resource Market

At present, China has accelerated the opening and sharing of data resources at the national and local levels. However, compared with countries with a high degree of data opening and sharing in the world, there are still many deficiencies, and the deployment of promoting the process of data opening and sharing should be accelerated.

First, establish the scope of open data sharing in the form of legislation. At present, China has not yet issued a complete relevant law on data sharing, and there are still no rules to follow in the current law. There is still a blank in legislation to clarify the scope of data sharing openness. According to the current situation, we can appropriately learn from the experience and practices of relevant countries, introduce special laws, regulations, and management regulations, or add relevant provisions to determine the scope of data sharing subjects within the current legal framework.

Second, build a unified data open platform to improve the quality and level of data open sharing. Relying on the advantages of national technical resources, I build a unified data open sharing platform, encourage the access of local data open sharing platforms at all levels, promote the standardized integration and aggregation of national data resources, and provide strong technical support to improve the open quality of public data resources.

Third, encourage enterprises with data resources and technical advantages to explore new models of data sharing. For enterprises that already have many data resources and have advantages in developing data resources, the state should appropriately encourage them to explore new data open sharing modes, promote good practice modes, promote enterprises to open data beneficial to social management to government departments and create a good atmosphere for social data sharing.

### 6.2. Strengthen Data Quality Management and Fully Realize Data Value

Data quality management affects data availability and is also a key factor in data transaction and circulation. Nonstandard data quality management and no guarantee of data quality are important reasons why it is difficult to establish the trust relationship between the supplier and the demander in data exchange. At the same time, unclear requirements for public data quality will also greatly reduce the effectiveness of public data open sharing.

Clarify the requirements for the quality of public data. China has clarified the quality requirements of public data in the unified public data opening legislation. At the same time, the regulatory authorities have made specific provisions on the data quality management of the industry and provided a reference for the data quality management of private subjects.

By formulating standardized data quality standards, the data quality requirements are further refined in the data development process and by using specific scenarios of transaction circulation. This effectively promotes the realization of the data standardization process and creates favorable conditions for the efficient flow of data in the factor market.

Build a third-party certification mechanism for data quality, and an authoritative third-party will evaluate and certify the industry data quality, encourage enterprises to do a good job in data quality management, and provide an effective guarantee for building the trust relationship between the supplier and the demander of the data resources market.

### 6.3. Promote the Transaction Flow of Data Resources and Activate the Data Resource Market

Promoting data transaction circulation is the key action to cultivate the data resources market and an important premise for data to flow in the market as a social factor of production and produce greater value.

Firstly, we need to speed up the improvement of relevant legislation to provide a legal basis for the circulation of data transactions. In the data related legislation, it is necessary to confirm the legal data transaction circulation mode and provide a clear data scope that prohibits or restricts the transaction circulation to clear the obstacles for the transaction circulation of data according to law and regulations.

At the same time, it is suggested that the regulatory authorities should issue guidance documents for inter-enterprise data circulation cooperation, consider the foreign experience and combining with the development practice of the industry, and issue relevant research report guidelines or guidelines for inter-enterprise data circulation innovation mode, contract model, and legal liability, so as to promote and standardize inter enterprise data circulation and to drive the efficiency of data circulation in the whole society.

Finally, a complete set of solutions for data circulation in various industries should be established, and the process of data circulation among enterprises in different business scenarios should be completed in the constructed industry data circulation space to further promote the realization of safe and efficient data circulation among enterprises.

### 6.4. Strengthen Data Security and Effectively Avoid Risks in the Cultivation of Data Resource Market

First, speed up relevant legislation for key data risk. Drawing on the experience of other countries in data security legislation, China should speed up the construction of relevant provisions on data disclosure notification, further clarify the trigger conditions of data disclosure notification, the specific requirements for notification and relevant legal responsibilities; build a scientific and effective data security risk prevention system; promote the establishment of laws and regulations related to information privacy protection; clarify the rights of individuals to their personal information; the obligations of enterprises to protect personal information.

The second is to build and improve the data law enforcement mechanism, clarify the division of responsibilities of different responsible departments in data risk regulation, establish an efficient law enforcement mechanism among departments, and make full use of new technologies, such as cloud computing, AI, and blockchain, improve the level and efficiency of data law enforcement, focus on data security management and personal information protection, and strengthen law enforcement. Provide guarantee for the orderly operation of data resources market.

Third, carry out in-depth research on emerging problems, such as data monopoly, and learn from foreign experience in dealing with new problems such as data monopoly and data unfair competition. Combining with industrial development practice, China should strengthen the research on new problems such as data monopoly, timely formulate relevant rules, revise existing laws and regulations, and strengthen the regulation of data monopoly risk.

## Figures and Tables

**Table 1:** Digital economy ranking of major countries in 2020.

Ranking	Country	The scale of the digital economy (USD 100 million)
1	U.S.A	135597
2	China	53565
3	Germany	25398
4	Japan	24769
5	U.K	17884
6	France	11870
7	Republic of Korea	8578
8	India	5419
9	Canada	4365
10	Italy	3775

DS: white paper on global digital economy, China Academy of ICT.

**Table 2:** DEDI index system in 2020.

DEDI			
*Basic indicators*			
Traditional digital infrastructure	Number of 4G users	New digital infrastructure	Bidding quantity of data center
	4G average download rate		Bidding amount of data center
	Number of fixed broadband users		Number of 5g pilot cities
	Fixed broadband average download speed		IPv6 ratio
	Internet penetration		
	Number of pages		
	Number of domain names		

*Industrial indicators*			
Industrial scale	Total output value of computer, communication, and other electronic equipment manufacturing industry	Industrial subject	Brush collar of main board listed enterprises in ICT field
	Total output value of information transmission, software, and information technology services		Number of top 100 internet enterprises
	Total telecom services		Number of unicorn enterprises

*Joint indicators*			
Integration of industry and information technology	Integration level of industry and informatization	Agricultural digitization	Number of digital rural innovation projects
	Digital level of production equipment		Number of Taobao villages
	Popularity of digital R&D and design tools	Digitalization of service industry	Number of third-party party payment financial licenses
	Proportion of applied E-commerce		E-commerce transaction volume
	Number of enterprises with high integration level		Number of internet hospitals
	Numerical control level of key processes		Number of national informatization education demonstration areas
			Number of smart parks

*Environmental indicators*			
Government new media	Number of government websites	Government data governance	Number of government data governance platform projects
	Number of social media accounts of government agencies		Government data platform construction fund investment and impetus
Government online services	Maturity of online handling of government online services		Construction of government data open platform above provincial level
	Online service efficiency of government online service		

DS: CCID consultant 2020.09.

**Table 3:** GDP and DEDI value from 2017Q4 to 2021Q3.

	GDP (RMB100 mn)	DEDI
2017Q4	235428.7	18.4
2018Q1	202035.7	18.5
2018Q2	223962.2	19.6
2018Q3	234474.3	20.5
2018Q4	258808.9	21.1
2019Q1	217168.3	21.6
2019Q2	241502.6	22.9
2019Q3	251046.3	24.0
2019Q4	276798	25.0
2020Q1	205727	24.1
2020Q2	248985.1	26.6
2020Q3	264976.3	27.5
2020Q4	296297.8	29.6
2021Q1	249310.1	29.7
2021Q2	282857.4	31.1
2021Q3	290963.8	31.8

DS: China National Bureau of Statistics and CCID consultant 2021.

**Table 4:** DEDI scores by province and region.

Ranking	Area	DEDI
1	Guangdao	65.3
2	Beijing	55
3	Jiangsu	52.2
4	Zhejiang	51.5
5	Shanghai	45.5
6	Shandong	42.8
7	Fujian	38.6
8	Sichuan	35.6
9	Henan	35
10	Hubei	32.5
11	Hebei	29.4
12	Hunan	29.4
13	Anhui	29.3
14	Chongqing	28.8
15	Jiangxi	28.5
16	Shanxi	26.3
17	Guangxi	26.2
18	Tianjin	24.9
19	Guizhou	24.7
20	Liaoning	23.5
21	Yunnan	21.3
22	Shanxi	21.1
23	Heilongjiang	20.5
24	Gansu	19.3
25	Neimenggu	18.9
26	Xinjiang	18.1
27	Hainan	17.8
28	Jilin	17.4
29	Ningxia	17.1
30	Qinghai	13.8
31	Xizhang	8

DS: CCID consultant 2020.

**Table 5:** Granger causality test results of the National Bureau of Statistics and CCID consultants in 2021.

Null hypothesis:	Obs	*F*-statistic	Prob.
*Y* does not Granger cause *X*	14	5.30381	0.0301
*X* does not Granger cause *Y*		5.60908	0.0262

**Table 6:** Government data service indicators of main areas of China.

	Government data service indicators (average is 31.4)
>31.4	Guangdong Zhejiang Shanghai Jiangsu Beijing
≈31.4	Guizhou Anhui Fujian Sichuan Hubei Hebei Henan Chongqing Hainan
<31.4, >30	Guangxi Tianjin Ningxia Yunnan Jiangxi Inner Mongolia Liaoning Shandong Heilongjiang Qinghai
<30	Jilin Shanxi Gansu Xinjiang Tibet Shaanxi

**Table 7:** Government data governance indicators of main areas of China.

	Government data governance indicators (average is 31)
>31	Beijing Guangdong Guizhou Shanghai Fujian
≈31	Shandong Ningxia Tianjin Guangxi Hainan Zhejiang Shaanxi Henan Jiangxi Chongqing
<31, >15	Jiangsu Gansu Hubei Yunnan Sichuan Hebei Xinjiang Hunan Inner Mongolia Heilongjiang
<15	Qinghai Anhui Shanxi Liaoning Jilin Tibet

## Data Availability

The research data used to support the findings of this study are included within the article.
